# Supplementary material for: HIVprotI: an integrated web based platform for prediction and design of HIV proteins inhibitors
Source: J Cheminform. 2018 Mar 9;10:12. doi: 10.1186/s13321-018-0266-y (PMC5845081; doi:10.1186/s13321-018-0266-y)
Supplement: Supplementary file 2 — Additional file 2. Source code of HIVProtI web server. [file 13321_2018_266_MOESM2_ESM.zip › HIVprotI_Source-code/hivprotI/weka-3-6-6/doc/weka/gui/SaveBuffer.html]

SaveBuffer


---


|  |  |  |
| --- | --- | --- |
| **Overview** \| **Package** \| **Class** \| **Tree** \| **Deprecated** \| **Index** \| **Help** \| **Weka's home** | |  |
| **PREV CLASS**   **NEXT CLASS** | **FRAMES**    **NO FRAMES**     **All Classes** |  |
| SUMMARY: NESTED \| FIELD \| CONSTR \| METHOD | DETAIL: FIELD \| CONSTR \| METHOD |  |


---


## weka.gui Class SaveBuffer

```
java.lang.Object
  weka.gui.SaveBuffer
```

---

``` public class SaveBuffer extends java.lang.Object ```

This class handles the saving of StringBuffers to files. It will pop
up a file chooser allowing the user to select a destination file. If
the file exists, the user is prompted for the correct course of action,
ie. overwriting, appending, selecting a new filename or canceling.

**Version:**
:   $Revision 1.0 $

**Author:**
:   Mark Hall (mhall@cs.waikato.ac.nz)

---

**Constructor Summary**

`SaveBuffer(Logger log,
java.awt.Component parent)`
  
          Constructor


| **Method Summary** | |
| --- | --- |
| `static void` | `main(java.lang.String[] args)`             Main method for testing this class |
| `boolean` | `save(java.lang.StringBuffer buf)`             Save a buffer |

**Methods inherited from class java.lang.Object**

`equals, getClass, hashCode, notify, notifyAll, toString, wait, wait, wait`

**Constructor Detail**

### SaveBuffer

```
public SaveBuffer(Logger log,
                  java.awt.Component parent)
```

:   Constructor

    **Parameters:**: `log` - the logger to send messages to: `parent` - the parent component will be requesting a save


**Method Detail**

### save

```
public boolean save(java.lang.StringBuffer buf)
```

:   Save a buffer

    :   **Parameters:**: `buf` - the buffer to save **Returns:**: true if the save is completed succesfully

---


### main

```
public static void main(java.lang.String[] args)
```

:   Main method for testing this class


---


|  |  |  |
| --- | --- | --- |
| **Overview** \| **Package** \| **Class** \| **Tree** \| **Deprecated** \| **Index** \| **Help** \| **Weka's home** | |  |
| **PREV CLASS**   **NEXT CLASS** | **FRAMES**    **NO FRAMES**     **All Classes** |  |
| SUMMARY: NESTED \| FIELD \| CONSTR \| METHOD | DETAIL: FIELD \| CONSTR \| METHOD |  |


---
